# Complex Effect of Concrete Composition on the Thermo-Mechanical Behaviour of Mass Concrete

**DOI:** 10.3390/ma11112207

**Published:** 2018-11-07

**Authors:** Barbara Klemczak, Maciej Batog, Zbigniew Giergiczny, Aneta Żmij

**Affiliations:** 1 Faculty of Civil Engineering, Silesian University of Technology, Gliwice 44-100, Poland; Zbigniew.Giergiczny@polsl.pl (Z.G.); Aneta.Zmij@polsl.pl (A.Z.); 2 Górażdże Cement S.A., Chorula 47-316, Poland

**Keywords:** mass concrete, early age properties, low clinker cement, aggregate, hardening temperature, mechanical properties, shrinkage, creep, early age stresses

## Abstract

The current work presents the complex investigation of the influence of cement and aggregate type on the thermo-mechanical behavior of mass concrete. Six types of cement with different amounts of non-clinker constituents and four types of aggregates are used in experimental tests. Particular attention was given to the low clinker cements with high amounts of siliceous fly ash and ground blast furnace slag. The experimental research covered the determination of thermal, mechanical, and rheological properties of early age concrete with different constituents. Experimental results have been used both to validate the numerical model and analysis of exemplary foundation slab. The results confirm the importance of the concrete mix composition and it has been shown that the early-age volume deformation and possible cracking is the result of the concerted action of thermal and mechanical properties of concrete. The obtained results indicate granite as the best aggregate for mass concrete. Considering the type of cement, much better behaviour of mass concrete has been noted for cements with fly ash and composite cements containing both fly ash and slags than cements only with slag.

## 1. Introduction

The temperature rise during cement hydration is still an open problem in the construction of mass concrete structures. Due to the considerable thickness of such structures, the amount of heat generated is significant and the core of the concrete member reaches particularly high temperatures, which is also higher than the temperature of its surface layers. Such non-uniform temperature distribution and its variation in time induces thermal strains. Additionally, shrinkage strains are also observed in early age concrete because of the hydration process and moisture exchange with the environment. Consequently, stresses arise in the concrete structure, which, if not properly controlled, can result in cracking, affecting service life of the structure. This early age cracking of thermal and shrinkage origin may initiate corrosion of reinforcement. Moreover, at a later age, the crack width may increase due to environmental and mechanical loads.

The size of early age thermal-shrinkage volume changes and possible cracking depends both on material and technological factors. Among others, concrete mix composition, type of concrete constituents, concrete curing condition, the size of concrete volume, and structure restraint conditions can be specified here [1,2]. In this respect, the concrete’s composition has a particularly significant effect on the discussed volume changes [3,4,5,6]. Hence, one of the methods for reducing the negative effects of self-heating of mass structures is the use of cements with moderate or low heat of hydration. Thus, the most practical way to limit hydration heat and in consequence the hardening temperature is the replacement of Portland cement with some non-clinker constituents as siliceous fly ash (FA) or ground blast furnace slag (GBFS). Such a replacement creates a binder which produces much less heat [7,8]. There is no doubt that the drawback of cements with non-clinker constituents is the strength gain, which is slower at early ages and may lessen the load-bearing capacity of the structure [9,10,11]. The slower strength gains at early ages is explained by the slower pozzolanic reaction of the slag and fly ash compared to Portland clinker. However, it was proved that pozzolanic reactivity of slag and fly ash contributes to strength gain at later ages [9,10]. Experimental results in this field visibly show the crucial effect of the amount of non-clinker constituents in cement on concrete strength development [12].

However, the examination of early-age volume changes in mass concrete cannot be only limited to the hydration heat and strength development. As it was emphasized in [4,5,6] that all material properties affecting the early-age volume changes and arising stresses in concrete should be considered. Consequently, a compilation of parameters including rate of hydration heat, thermal conductivity, heat capacity, thermal dilation, elasticity modulus, tensile strength, and creep with respect to maturity time were affected strongly by the hardening temperature and should be taken into account. It can be noticed that many studies addressed the effect of different concrete constituents and describe properties of early age mass concrete in an independent manner without complex analysis of the thermo-mechanical behaviour of the real mass structure. Relatively rare works in this area usually refer to the experimental research of selected properties or validation of experimental tests performed on laboratory samples [4,5,6]. If a real structure is analysed, usually part of the input data is based on theoretical assumptions.

This paper is focused on the overall early age behavior of mass concrete structure made of concrete with different constituents. In particular, the influence of cement and aggregate type on the formation of hardening temperature, moisture loss, and stress development in early age concrete has been studied. The work consists of experimental and numerical study. In the experimental part, six types of cement with different amount of non-clinker constituents (fly ash and slag) and four types of aggregates (gravel, basalt, granite, and limestone) have been considered. Special attention was paid to the low clinker cements with high amount of siliceous fly ash and ground blast furnace slag. Therefore, cements containing even 68% of non-clinker constituents are used in concrete. The experimental research covered the determination of thermal (hydration heat, thermal conductivity and capacity, temperature development), mechanical (elasticity modulus, tensile, and compressive strength), and rheological (shrinkage and creep) properties of early age concrete with different constituents. First, the obtained experimental results have been used to validate the applied numerical model. Next, the analysis of mass foundation slabs has been performed. The main purpose of this analysis was to indicate that the early-age volume deformation and possible cracking is the result of the concerted action of many factors relating to thermal and mechanical properties of concrete.

## 2. Experimental Research

### 2.1. Materials

Two compositions of concrete mix with different cement and aggregate type were used in the experiments, as shown in Table 1. Type A concrete was applied for gravel and limestone aggregates while type B consisted of basalt and granite aggregate.

Six commercial cements with different content of Portland clinker, siliceous fly ash, and/or granulated blast-furnace slag were used for the experimental tests: Portland slag cement CEM II/B-S 32.5R, Portland-fly ash cement CEM II/B-V 32.5R, slag cement CEM III/A 32.5N-LH/HSR/NA, composite cement CEM V/A (S-V) 32.5R-LH/HSR/NA, special composite cement with high amount of non-clinker constituents VLH V/B (S-V), and CEM I 42.5R as a reference cement. The content of non-clinker constituents in the cement compositions varied from 27.1% to 67.7%, as listed in Table 2. 

### 2.2. Scope and Testing Methods

The research program covered the following tests: physical and mechanical properties of cements, hydration heat of cements, compressive and tensile strength of concretes, elasticity modulus of concrete, concrete hardening temperature, thermal conductivity and heat capacity of concretes, concrete shrinkage, and creep. Some results of this extensive research have been already presented, including physical and mechanical properties of cements [3,7], hydration heat evolution under adiabatic conditions and isothermal conditions at 20 °C and 50 °C [3,7], compressive strength development of concrete with different cements [3,9], hardening temperature of concrete [3], thermal conductivity, and heat capacity [3]. Details of these tests and their results have been extensively described in previous works [3,7,9]. Not reported results are: development of compressive strength of concrete with different aggregate types, tensile strength development, Young’s modulus development, shrinkage and creep development of concrete with different constituents.

Concrete compressive strength was tested after 1, 2, 7, 28, 56, and 90 days of concrete curing. Concrete cubes 150 mm × 150 mm × 150 mm were used according to the procedure enclosed in EN 12390-3 [14]. Tensile strength was determined in bending tests after 2 and 28 days. Samples with the dimensions 150 mm × 150 mm × 700 mm were used, and the testing procedure included in PN-EN 12390-5 [15] has been followed. The modulus of elasticity was determined after 2 and 28 days on cylindrical samples 150/300 mm in accordance with a test methodology described in ISO 1920-10 [16]. All specimens were stored in the mold for 24 h. Next, the demoulded specimens continued curing at a temperature of 18 °C (±2 °C) and 90% relative humidity. 

Shrinkage strains were measured on cuboidal samples with the dimensions 100 mm × 100 mm × 500 mm according to the methodology of PN-B-06714-23 [17], after 2, 7, 14, 28, 56, 90, 120, 150, 210, 270, and 360 days of concrete curing. Samples were stored for 24 h in a climate chamber at 18 °C (±2 °C) and relative humidity above 90%. Next, the demoulded samples were subjected to drying in laboratory conditions at 18 °C (±2°C) and 65–75% relative humidity. Similarly, concrete creep strains were measured on samples 100 mm × 100 mm × 500 mm. The same curing conditions were applied as for shrinkage tests. Next, the samples were subjected to a compressive force equal to 33% of the failure force obtained from 2-day concrete compressive strength tests. The creep strains were registered for 90 days.

The tests were performed on concretes with the constituents given in Table 1, considering the different type of aggregate and cement. Tests of concrete with gravel aggregate were made with the use of 6 types of cement. Compressive and tensile strength, modulus of elasticity, and shrinkage deformations of concretes with basalt, granite, and limestone aggregates were tested with the use of 3 types of cement: CEM I 42.5R, CEM III/A 32.5N-LH/HSR/NA, and CEM V/A (SV) 32.5R-LH/HSR/NA. Creep tests were performed for concrete with gravel aggregate and the six types of cements described in Table 2.

### 2.3. Test Results

**Figure 1:**
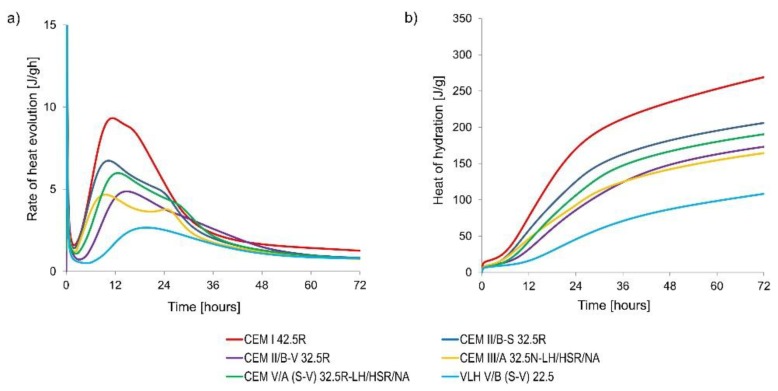
Rate of heat evolution (**a**) and heat of hydration (**b**) under isothermal conditions at 20 °C.

As it has been mentioned in the previous section, some results of this extensive research have been already presented [3,7,9]. Nevertheless, for completeness of the study and later results of the numerical analysis, some of them are concisely quoted in Figure 1 and Table 3 and Table 4.

**Figure 2:**
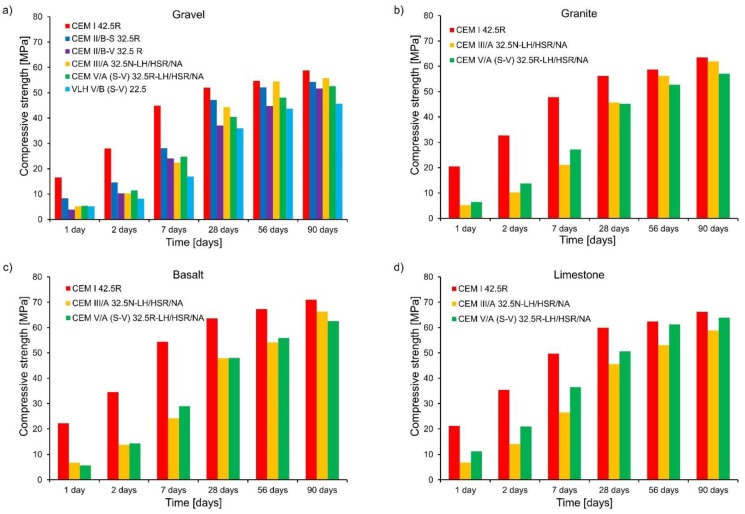
Development of concrete compressive strength: (**a**) gravel, (**b**) granite, (**c**) basalt, and (**d**) limestone.

**Figure 3:**
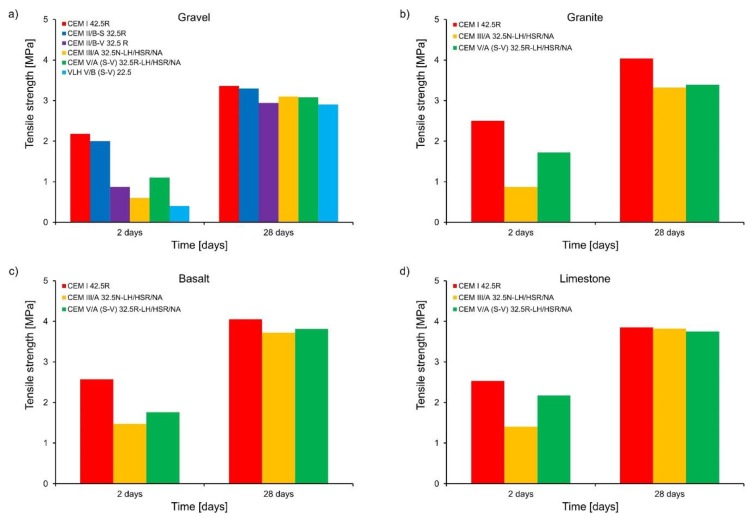
Development of concrete tensile strength: (**a**) gravel, (**b**) granite, (**c**) basalt, and (**d**) limestone

The results of compressive and tensile strength determined for concrete samples with different types of cement and aggregates are shown in Figure 2 and Figure 3, respectively. The following summary of these results can be written:

- Significantly lower early age compressive and tensile strength are observed in concretes made of cements with non-clinker constituents. These values were lower as the addition of non-clinker constituents increased. However, the non-clinker constituents in cement favorably improved the compressive strength of concrete after 28 days. It is also worth noting that the tensile strength of concretes with cement containing fly ash (CEM II/B-V 32.5R) is greater than that of concrete with cement containing slag (CEM III/A 32.5N-LH/HSR/NA), while both cements revealed similar amount of heat evolved during hydration (Figure 1). It confirms some other results [18] indicating that the use of cements with fly ash improves the tensile strength of concrete;
- Concrete containing granite, basalt, or limestone aggregates achieved higher average compressive strength than concrete with gravel aggregate. The highest average compressive strength was obtained for concrete with basalt aggregate and in the early age period it was even 33.7% higher than for concrete with gravel aggregate. Concrete with granite or limestone aggregate was characterized by early age strength comparable to concrete with basalt aggregate;
- Similarly, the average tensile strength of concrete containing granite, basalt, or limestone aggregates was higher than those with gravel aggregate. In the case of concrete with cement CEM I 42.5 R after 2 days, they were higher by 14.7% to 17.9%. However, in the case of CEM III/A 32.5N-LH/HSR/NA, the average tensile strength was higher by 45.0 to 145.0% in comparison to the gravel aggregate, and in the case of cement CEM V/A (SV) 32.5R -LH/HSR/NA, tensile strength was higher by 56.4 to 97.3% compared to concretes with gravel aggregate. After 28 days, the tensile strength of concretes with basalt, granite, or limestone aggregate was higher only by 23.7%.

**Figure 4:**
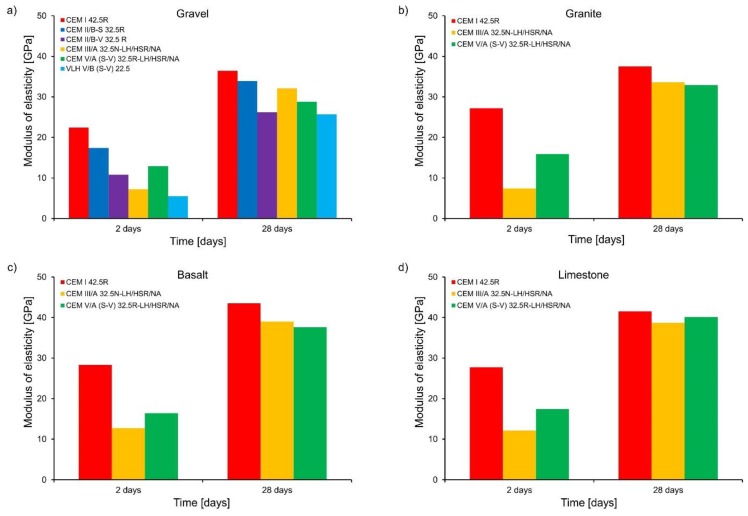
Modulus of elasticity of concrete: (**a**) gravel, (**b**) granite, (**c**) basalt, and (**d**) limestone.

The obtained values of modulus of elasticity are plotted in Figure 4. The highest early age modulus of elasticity was obtained for concrete with Portland cement CEM I 42.5R. Concretes with cements containing non-clinker components were characterized by much lower modulus of elasticity at early age. Reduction of the modulus of CEM I 42.5R concrete ranged from 22.3% (CEM II/B-S 32.5 R) to 75.4% (VLH V/B (S-V) 22.5). Thus, the following relationship can be stated: the greater the amount of non-clinker components in the cement, the lower the early age modulus. The elastic modulus increased significantly after 28 days of hardening for these cements, however, the obtained values are lower than in the reference concrete with Portland cement CEM I 42.5 R, respectively, from 7 to 12% (in the case of concrete with CEM II/BS 32.5R or CEM III/A 32.5N-LH/HSR/NA) and from 19 to 29% (for concrete with CEM V/A (SV) 32.5R-LH/HSR/NA, CEM II/BV 32.5R and VLH V/B (SV) 22.5). Concrete containing granite, basalt, or limestone aggregates reached higher modulus of elasticity than concretes with gravel aggregate. The highest values are registered both after 2 days and 28 days of concrete curing for concrete with basalt aggregate concrete.

**Figure 5:**
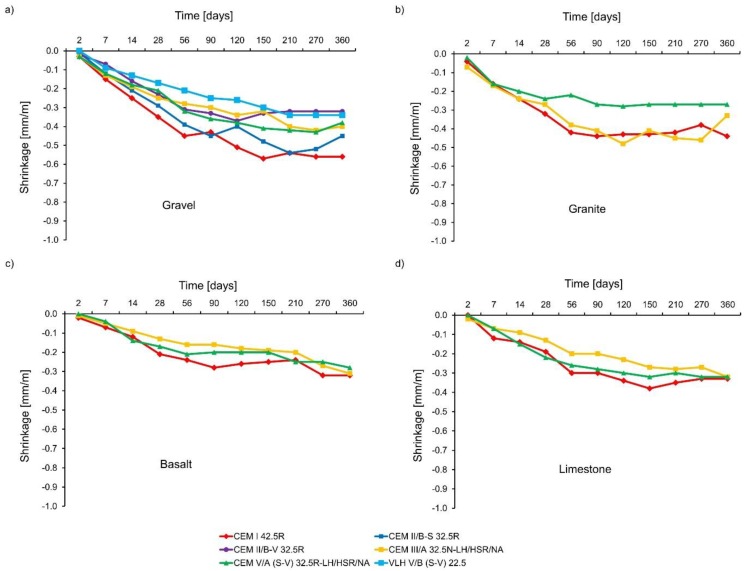
Concrete shrinkage strains: (**a**) gravel, (**b**) granite, (**c**) basalt, and (**d**) limestone.

The results of shrinkage tests are plotted in Figure 5. It has been found that the use of cements with non-clinker main components in the concrete composition reduces the shrinkage of concrete. The following relationship can be also distinguished—the higher the amount of non-clinker main components is applied; the smaller the concrete shrinkage is. However, this reduction in shrinkage strains is not in direct proportion to the increase of non-clinker components used in the cement composition. It is worth adding that the samples had the same maturing conditions, so the differences in the shrinkage strains result mainly from different autogenous shrinkage. This is confirmed by similar shrinkage values for concretes with CEM II/B-V 32.5R, CEM III/A 32.5N-LH/HSR/NA, and CEM V/A (SV) 32.5R-LH/HSR/NA, which showed very similar amount of heat evolved during hydration (Figure 1). The use of granite, basalt, or limestone as aggregate reduces the shrinkage strains compared to gravel aggregate. The lowest shrinkage strains, both early and long-term, were registered in concrete with basalt aggregate.

**Figure 6:**
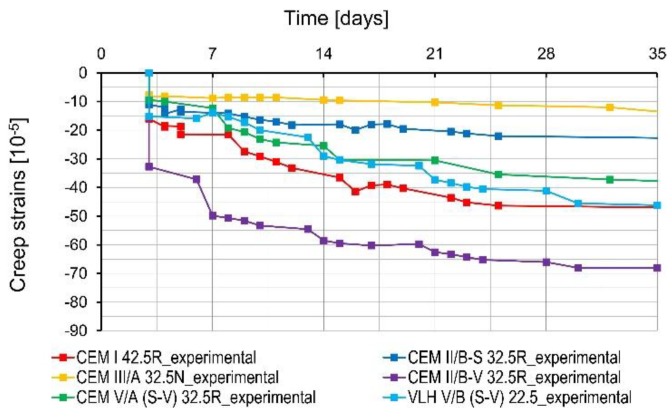
Concrete creep strains.

The results of the measured creep strains are depicted in Figure 6. The shrinkage has been subtracted from the creep measurement. Concrete with fly ash cement (CEM II/B-V 32.5 R) is characterized by the greatest creep strains in the early age period (up to 7 days), as well as in later period (7–28 days). The concrete creep of concrete with this cement is also higher than for concrete made of Portland cement CEM I 42.5 R (reference concrete). Similar results for concrete with fly ash cement are also mentioned in [10,18]. Concrete containing slag cement (CEM III/A 32.5N-LH/HSR/NA) as well as both slag and fly ash cement (CEM V/A (SV) 32.5R-LH/HSR/NA) showed the lowest creep strains among all tested concrete in the early period (up to 7 days). All concretes with cements containing only granulated blast furnace slag as a non-clinker component (CEM II/B-S 32.5 R and CEM III/A 32.5 N-LH/HSR/NA) were characterized by lower creep than the reference concrete, and the creep strains decreased with the increase of slag in cement content. Similar relationships are mentioned in [19]. In the case of concretes with CEM V/A (S-V) 32.5R-LH/HSR/NA and VLH V/B (S-V) 22.5, no relationship was found between the amount of non-clinker component and creep strains level—the obtained creep strains are at a similar level. Probably, it is the result of the opposite effect of silica fly ash and granulated blast furnace slag on the development of creep strains.

## 3. Comments on the Experimental Results Regarding the Most Favourable Early Age Behaviour of Mass Concrete

The best concrete for a massive structure would be the concrete with:

- small heat evolved during cement hydration (resulting in low hardening temperature),
- high thermal conductivity (resulting in fast heat dissipation from the structure),
- high heat capacity (resulting in lower hardening temperature),
- small coefficient of thermal expansion (resulting in lower deformation),
- small shrinkage (lowering the total early age deformation),
- small modulus of elasticity and high creep (decreasing of the arising stresses),
- high tensile strength (increasing the early age cracking resistance).

Referring the obtained experimental results to the above-mentioned beneficial properties, the following comments can be written:

- the use of cements with a large amount of non-clinker components in mass concrete is beneficial due to reduced hydration heat which decreases the hardening temperature and to a smaller elastic modulus which decreases the arising stresses; nevertheless, the tensile strength of such concrete is much lower and consequently the early age cracking resistance decreases,
- the use of cements with a large number of non-clinker components results also in lower shrinkage which is preferred in mass concrete,
- greater early age creep is beneficial in mass concrete as it reduces early age stresses, therefore, in this regard, it is better to use cements containing silica fly ash rather than cements containing ground blast furnace slag which showed lower creep,
- the effect of aggregate is particularly complex. Considering thermal conductivity, heat capacity and modulus of elasticity concrete with gravel aggregate has the best properties for mass concrete, but simultaneously, the thermal dilation of concrete with this aggregate is the largest [20,21]. In contrast, concrete containing granite, basalt, or limestone aggregates reached higher modulus of elasticity and lower thermal conductivity and heat capacity than concretes with gravel aggregate, but the thermal dilation of concrete, tensile strength, and shrinkage are more beneficial.

## 4. Thermo-Mechanical Analysis

### 4.1. Numerical Model

The original numerical model has been developed for the 3D analysis of early age concrete and it can be applied for reinforced concrete structures as foundation slabs and blocks, tank walls, and bridge abutments. The complex analysis of the mentioned structures is run in two stages. In the first stage, temperature and moisture development in time are determined. The original feature of this stage is the application of coupled equations of thermodiffusion. Next, thermal-shrinkage strains are calculated, and these results are used as an input for computation of stresses in the second stage of analysis. In analysis of early age stresses, the viscoelastic material model of ageing concrete is applied. The influence of elevated curing temperatures on concrete mechanical properties as well on the development of hydration heat is considered by introducing equivalent concrete age instead of the real age of concrete [7,22,23].

The model considers soil–structure interaction and appropriate technological conditions like layered casting, environmental conditions like ambient temperature and humidity, different initial temperature of the concrete layers, and the time of formwork removal. The full thermo-mechanical concrete properties are also considered. For practical application, original computers programs have been developed: TEMWIL for thermal–moisture analysis and MAFEM for stress analysis. Detailed description of the model and computer programs TEMWIL and MAFEM can be found in [22,23,24]. 

### 4.2. Validation of the Model

First, experimental results have been computer program TEMWIL used to validate the numerical model. Validation of the model covered the determination of hardening temperature of concrete and shrinkage and creep strains. Some considerations on the correct description of the development of mechanical properties and proper value of the activation energy of concretes with different constituents have been enclosed in [9,25] and were used in the analysis.

**Figure 7:**
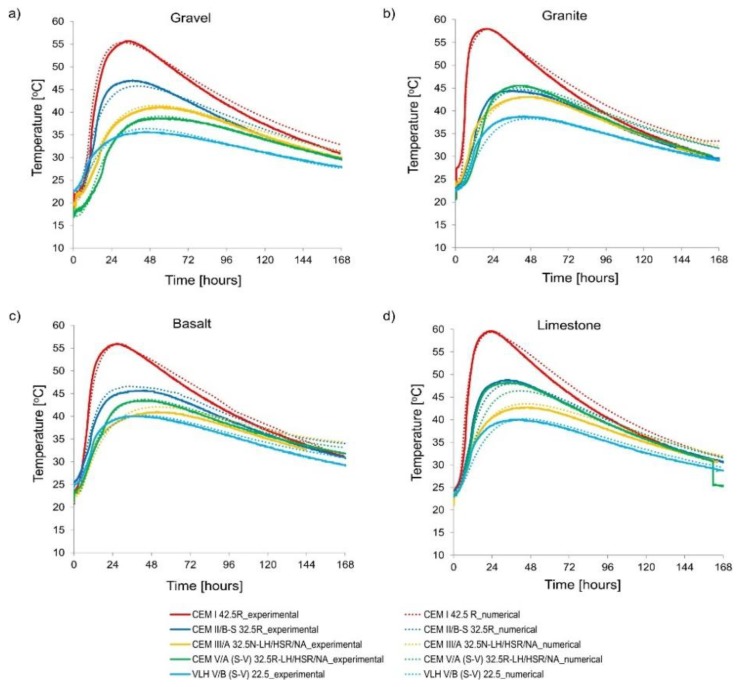
Experimental and numerical results of hardening temperature: (**a**) gravel, (**b**) granite, (**c**) basalt, and (**d**) limestone.

The results of numerical calculations and experimentally registered temperature of cubic specimens 40 cm × 40 cm × 40 cm [3] are depicted in Figure 7. In all cases, an acceptable agreement was achieved for temperature development in time. That proves the proper parameters of the model assumed in thermal analysis. The detailed values of thermal parameters are summarized in Table 5.

**Figure 8:**
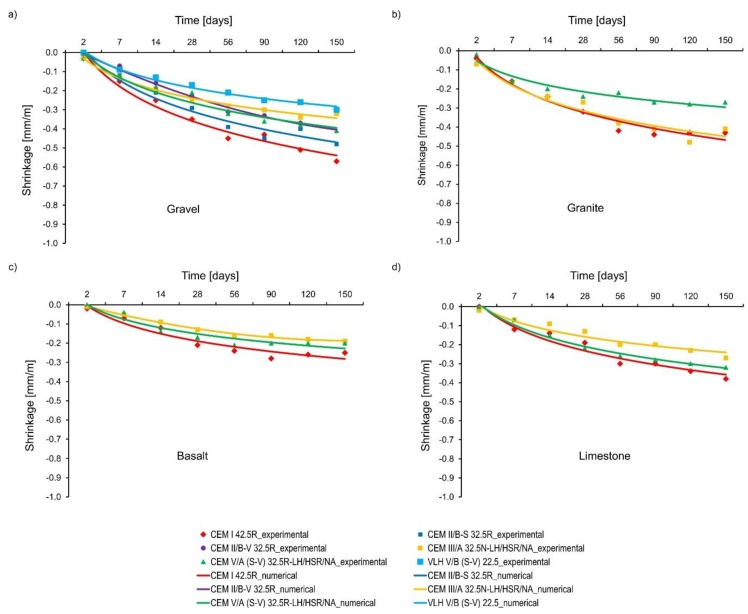
Experimental and numerical results of shrinkage strains.

In the validation of shrinkage tests, the best agreement between the results of calculations and the experiment was obtained for the following values assumed in the numerical model: moisture transfer coefficient equal to 2.78 × 10^−8^ m/s, coefficient of moisture dilatation *α_w_* = 0.001/0.0009/0.0006/0.0008 for gravel/granite/basalt/limestone. The other parameters were adopted in accordance with Table 5. Figure 8 compares experimentally-measured shrinkage with numerical results. It is worth noting that the differences in the shrinkage deformations for concretes with different cements result from the different autogenous shrinkage of these concretes, because the external conditions were the same for all samples. This effect is possible to simulate in the computer programme TEMWIL as the coupled equations of heat and mass transfer are used.

**Figure 9:**
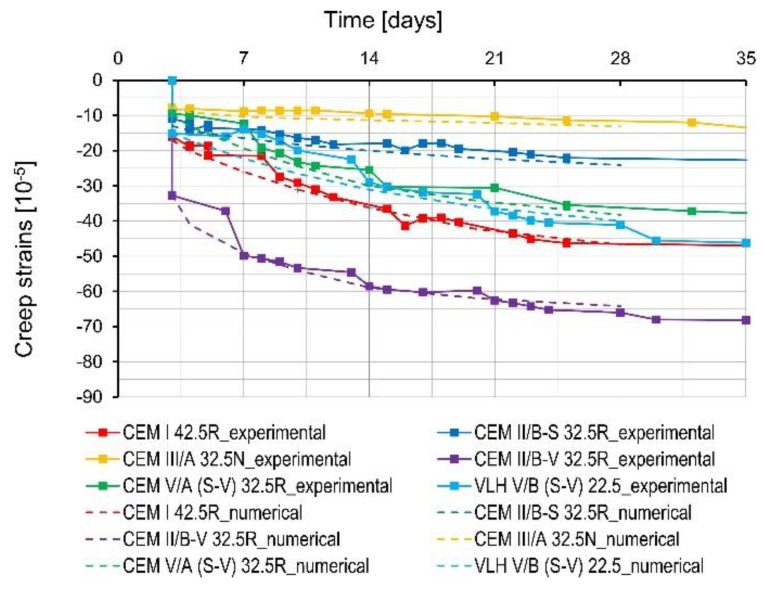
Experimental and numerical results of creep strain.

A concrete creep test was also simulated. The calculations considered the development of the mechanical properties of concretes determined during experimental investigations and other experimental data. Good agreement between the results of numerical calculations and the results of experimental tests can be noticed in Figure 9.

### 4.3. Analysis of Massive Foundation Slabs

#### 4.3.1. Object and Scope of Analysis

**Figure 10:**
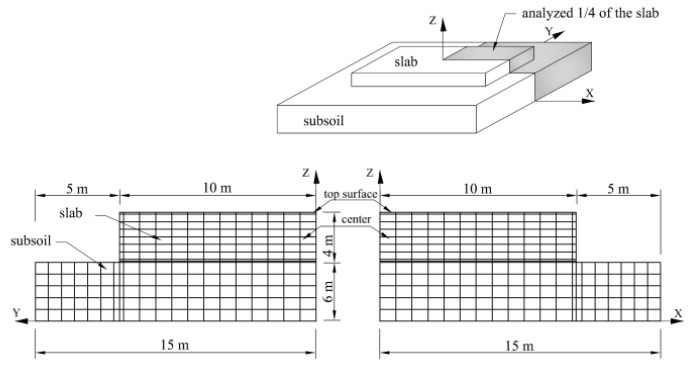
Assumed finite element mesh in the slab.

Finally, the complex influence of concrete constituents on the thermo-mechanical behavior has been investigated in mass foundation slabs with the base dimensions 20 m × 20 m and 4 m thickness. The finite element mesh of the slab is shown in Figure 10. Only a quarter of the slab is modelled because of its symmetry. The data for the numerical study was taken on the base of experimental tests described in former sections. The same data given in Table 5 was applied with the exception of the thermal transfer coefficient equal to 6.0 W/(m^2^K) (top surface without any protection) and equal to 3.5 W/(m^2^K) (side surfaces with plywood). Similarly, moisture transfer coefficient was assumed to 2.78 × 10^−8^ m/s at top surface and to 0.18 × 10^−8^ m/s at side surfaces. Ambient and initial temperature of the slab were taken as 20 °C. All computational cases are listed in Table 6.

#### 4.3.2. Results and Discussion

In thick foundation slabs, the self-induced stresses resulting from non-uniform volume changes in a cross section are predominant. These stresses have a distinctive distribution during concrete hardening. In the heating phase, the tensile stresses exist in surface layers, while compressive stresses are observed inside the slab. Next, an inversion of the stresses occurs in the cooling phase. In this phase, the tensile stresses are observed in the core of the slab and compressive stresses exist in the surface layers [23]. Thus, in foundation slabs, the early age cracks are usually observed on the top surface in the heating phase, because of the tensile stresses existing in surface zones of the slab. That is why the results regarding the development of stresses has been limited to the presentation of tensile stresses on the surface of the slabs. Similarly, in analysis of hardening temperature, the results have been discussed in relation to the recommended maximum temperature difference between the core and the surface of a concrete structure equal to 15–20 °C [1,2,19].

##### Influence of cement type

**Figure 11:**
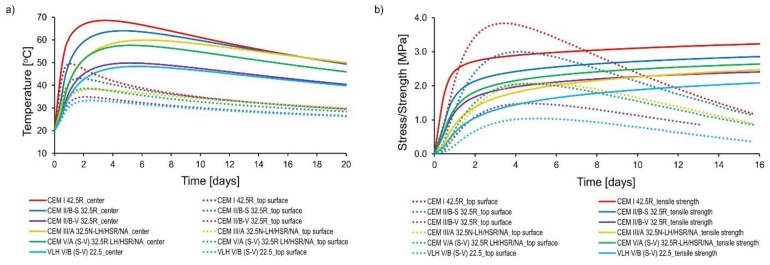
The results of numerical study of slabs with different cement type: (**a**) temperature development, (**b**) tensile stress and strength development.

**Figure 12:**
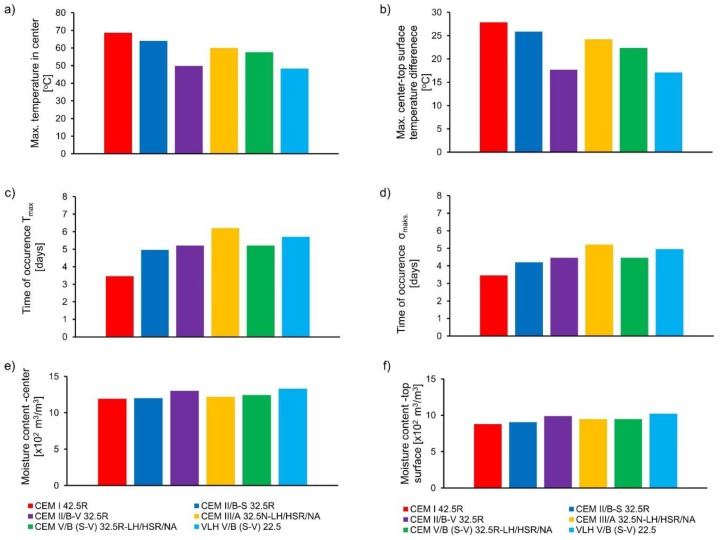
The results of numerical study of slabs with different cement type: (**a**) maximum hardening temperature T_max_, (**b**) maximum center-top surface temperature difference, (**c**) time of occurrence T_max_, (**d**) time of occurrence of maximum tensile stress s_max_, (**e**) moisture content at the center of the slabs, (**f**) moisture content at the top of the slabs.

The results of numerical analysis of massive foundation slabs with different cement types are presented in Figure 11 and Figure 12. The following remarks can be made from the above-mentioned figures:

- In case of cements: CEM II/B-V 32.5R and CEM II/B-S 32.5 R, which contain silica fly ash or granulated blast furnace slag of similar amount (about 30%), the higher performance of fly ash was observed. Slabs with CEM II/BV 32.5R cement were characterized by 14.2 °C lower core temperature in relation to CEM II/ BS 32.5R cement. The temperature difference between the core and the surface of the slabs made of CEM II/BV 32.5R did not exceed 20 °C despite the considerable thickness of the slab. Also, tensile stresses at the top surface are much lower than the current tensile strength of concrete. It is worth noting that concrete with CEM II/B-V 32.5R has a low modulus of elasticity and high creep, which is beyond the low hydration heat, also the reason for its low tensile stresses in comparison to CEM II/BS 32.5R,
- The use of CEM III/A 32.5N-LH/HSR/NA cement with a slag content of 58.9% does not guarantee early age cracking resistance of the slabs. In this case, the tensile stresses are greater than the tensile strength,
- In the case of using CEM V/A (SV) 32.5R-LH/HSR/NA (the content of non-clinker components was 37.8%), a decrease in maximum temperature and center-surface temperature difference was observed in the foundation slab. The mentioned values were at a level similar to that of CEM III/A 32.5N-LH/HSR/NA, in which the addition of the non-clinker component is much higher (58.9%). Tensile stresses are relatively high in relation to the tensile strength, but they have not exceeded it,
- The foundation slabs of concrete containing VLH V/B (S-V) 22.5 were characterized by the lowest maximum center temperature as well as the lowest center-surface temperature difference. This is due to the lowest heat of hydration of VLH V/B (S-V) 22.5 of all the analyzed cements. The values of the surface stress were halved in comparison to concrete with cement CEM I 42.5R. This was also due to the much lower modulus of elasticity of concrete with this cement.
- The increase in the amount of non-clinker main components in the cement affects the delay of the maximum temperature and maximum tensile strength occurrence. The following relationship is visible: the higher the content of the non-clinker component in the cement, the greater the delay in comparison to the reference concrete with Portland cement CEM I 42.5R. At the same time, it was not noticed that the type of non-clinker component influenced the discussed delay.
- It was observed that the type of cement affects the moisture content of concrete in the analyzed period. These differences are the result of the varying amount of water bound in the cement hydration process, which is a direct consequence of the different amount of heat released. It is worth noting that significantly greater moisture loss occurred on the surface of the analyzed slabs, which is consistent with the work [27], which states that considerable moisture loss and shrinkage appear in the surface layer of mass concrete but do not significantly affect the moisture content inside this structure.

##### Influence of aggregate type

**Figure 13:**
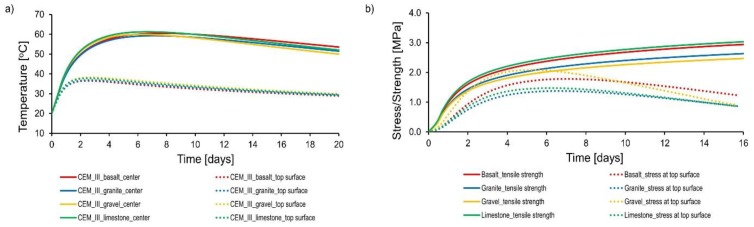
The results of numerical study of slabs with different aggregate type: (**a**) temperature development, (**b**) tensile stress and strength development.

**Figure 14:**
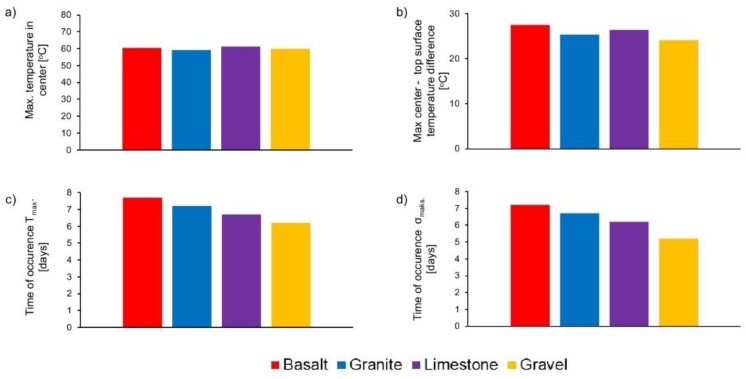
The results of numerical study of slabs with different aggregate type: (**a**) maximum hardening temperature T_max_, (**b**) maximum center-top surface temperature difference, (**c**) time of occurrence T_max_, and (**d**) time of occurrence of maximum tensile stress s_max_.

The results of numerical tests are presented in Figure 13 and Figure 14. First of all, it can be noticed that the type of aggregate does not significantly affect the temperature distribution in the foundation slab. The effect of the aggregate type on the maximum temperature of the core and the surface is not greater than 3 °C. Small differences in the maximum temperature reflect small differences in the heat capacity of concretes with these aggregates. Similar results were obtained during experimental research.

The difference in temperature between the core and the surface exceeded 20 °C in all slabs. Nevertheless, only in case of concrete with gravel aggregate were stresses exceeding the maximum concrete tensile strength observed. The reason is the low tensile strength and higher coefficient of thermal expansion of concrete (resulting from the presence of quartz in the gravel aggregate) compared to concretes with other aggregates. In this case, the lower elastic modulus of concrete with gravel aggregate did not balance the larger thermal deformations. Thus, the complex effect of the concrete mix composition on the thermo-mechanical behavior of massive structures is particularly visible here.

The type of aggregate used in the concrete composition affects the time of the occurrence of maximum temperatures and maximum tensile stresses. The shortest time was obtained for concrete with gravel aggregate. In the case of concretes with limestone, granite, or basalt aggregates, the maximum temperature occurred 12, 24, and 36 h later, respectively, in comparison to concrete with gravel aggregate. This is due to the different thermal conductivity of these concretes. Similarly, the maximum tensile stresses occurred 24, 36, and 48 h later than for concrete with gravel aggregate.

The use of gravel aggregate in the concrete is the least favorable due to the values of maximum stresses in the analyzed foundation slabs, and due to the relatively short time of their occurrence. Regarding the mentioned properties, the granite aggregate in the concrete composition seems to be the most optimal. The analyzed foundation slabs made of this concrete were characterized by the lowest maximum temperature and tensile stresses. Occurrence of maximum temperature and maximum stresses is also delayed. The use of basalt and limestone aggregates is also more advantageous than gravel aggregate.

## 5. Conclusions

This research presents the results of experimental and numerical studies on the overall early-age properties and behavior of mass concrete structures. Since concrete is a heterogeneous material whose behaviour depends on the properties of its constituents, the cement and aggregate type has been studied. Although in some works [28,29] the properties of concrete are tested and described at the microstructure level, the research results published in this article may also be useful. 

The use of cements with non-clinker main components in mass concrete, especially with a large number of them (>35%), reduces the maximum hardening temperature and generated stresses and delays the time of their occurrence. The presented results show that the use of cements with addition of siliceous fly ash in concrete is more advantageous than cements containing granulated blast furnace slag in mass concrete. Experimental tests and numerical analysis of foundation slabs made of concretes containing cements with silica fly ash showed that the maximum temperature and tensile stress are lower, which results from the lower hydration heat of these cements, lower elasticity modulus of concrete, less shrinkage, and greater creep compared to concretes with cements containing granulated blast furnace slag.

It should be noted that the conclusions regarding the benefits of using cement with non-clinker main components in massive structures are not new. However, they were mainly related to limiting the hardening temperature of concrete. Furthermore, the composition of the concrete was usually determined in terms of limiting the maximum temperature and ensuring adequate compressive strength of the concrete without a comprehensive analysis of all its properties. Similarly, other beneficial properties of such cements (low shrinkage, high creep, low modulus of elasticity) are not raised very often.

Based on the analyses and experimental tests, the tested cements may be ordered from the most to the least favorable based on early thermal behaviour in mass concrete: VLH V/B (SV) 22.5; CEM II/BV 32.5R; CEM V/A (SV) 32.5R-LH/HSR/NA; CEM III/A 32.5N-LH/HSR/NA; CEM II/BS 32.5R; CEM I 42.5R. Therefore, considering the type of cement, much better behaviour of mass concrete has been noted for cements with fly ash and composite cements containing both fly ash and slags than cements with slag only.

It is also worth mentioning that curing temperature exceeding 65–70 °C can lead to delayed ettringite formation (DEF) and consequently to possible later damage of the concrete. In this respect, as it was indicated in [30,31] that the use of silica fly ash or granulated blast furnace slag as a cement component has the following additional advantages:

- silica fly ash above 15% reduces DEF, and with content above 30%, DEF does not occur,
- granulated blast furnace slag in the amount of 17.5% limits DEF and is preventative above 25–30%.

The significant influence of aggregate type on the stress development in the mass structures has also been highlighted. The conducted analysis revealed that the gravel aggregate, consisting mainly of quartz, can be considered as the worst aggregate, while granite aggregate may be treated as the best. Therefore, the proper selection of aggregate is also important for reducing early-age cracking in mass concrete. Simultaneously, the effect of the aggregate is very complex and all thermal and mechanical properties should be carefully considered.

Thus, the results confirm that the early-age volume deformation and possible cracking is the result of the concerted action of thermal and mechanical properties of concrete whose depend primarily on the concrete constituents. 

Finally, despite the fact that the research program was relatively extensive, it requires continuation by others in the field of tensile creep testing, as concrete exhibits higher creep under tension [32,33] and the early-age tensile stresses determined under the assumption of symmetrical creep may be overestimated. Similarly, the tensile strength of mass concrete should be investigated due to the published research indicating its reduction in large structures [34].

## Figures and Tables

**Table 1:** Concrete mix composition.

Component	Content, kg/m^3^	
	Gravel^1^ and Limestone	Basalt and Granite
Cement (6 types)	300	
Water	150	
Sand 0/2 mm	583	
Aggregate 2/8 mm	427	-
Aggregate 8/16 mm	389	-
Aggregate 16/31.5 mm	544	-
Aggregate 2/8 mm	-	427
Aggregate 8/16 mm	-	389
Aggregate 16/22.5 mm	-	544

^1^ the aggregate described as a gravel consists mainly of quartz.

**Table 2:** Composition of cements.

Cement Type	Non-Clinker Constituents, %			
	Portland Clinker	Ground blast Furnace Slag (GBFS)	Siliceous Fly Ash (FA)	Minor Constituents ^1^
CEM I 42.5R	95.7	-	-	4.3
CEM II/B-S 32.5R	68.3	27.1	-	4.6
CEM II/B-V 32.5R	65.7	-	29.9	4.4
CEM III/A 32,5N-LH/HSR/NA^2,3^	41.1	58.9	-	-
CEM V/A (S-V) 32.5R-LH/HSR/NA^2,3^	62.2	18.2	19.6	-
VLH V/B (S-V) 22.5	32.3	34.4	33.3	-

^1^ Limestone with 98.2% of CaCO_3_ and TOC = 0.07%; ^2^ HSR—sulphate resistant cement acc. to Polish Standard PN-B-19707:2013 [13]; ^3^ NA—low alkaline cement acc. to Polish Standard PN-B-19707:2013 [13].

**Table 3:** Influence of cement and aggregate type on concrete thermal conductivity [3].

Thermal Conductivity [W/(m·K)]				
Cement Type	Aggregate Type			
	Gravel	Basalt	Granite	Limestone
CEM I 42.5R	2.43	1.84	2.01	2.03
CEM III/A 32.5N-LH/HSR/NA	2.35	1.70	1.75	1.99
CEM V/A (S-V) 32.5R-LH/HSR/NA	2.28	1.71	1.98	1.96

**Table 4:** Influence of cement and aggregate type on concrete heat capacity [3].

Heat Capacity [10^6^·J/(m^3^·K)]				
Cement Type	Aggregate Type			
	Gravel	Basalt	Granite	Limestone
CEM I 42.5R	1.72	1.83	1.81	1.71
CEM III/A 32.5N-LH/HSR/NA	1.68	1.77	1.74	1.76
CEM V/A (S-V) 32.5R-LH/HSR/NA	1.61	1.76	1.77	1.67

**Table 5:** Basic data for numerical validation.

Properties/Coefficient	Value
Concrete composition	acc. to Table 1
Thermal conductivity	$\lambda_{0}$ acc. to Table 3, with consideration of hydration rate α: $\lambda =\lambda_{0}\left(2-\alpha \right)$ [26]
Specific heat	$c_{b0}$ acc. to Table 4, with consideration of hydration rate α: $c_{b}=c_{b0}\left(1.25-0.25\alpha \right)$ [26]
Coefficient representing the influence of the moisture concentration on heat transfer	9.375 × 10^−5^ m^2^K/s
Thermal transfer coefficient	0.248 W/(m^2^K)–all surfaces (150 mm insulation [3])
Heat of hydration	on the basis of Figure 1 and results reported in [7,25], with the consideration of concrete hardening temperature by means of an equivalent concrete age
Water-cement proportionality	0.3 × 10^−9^ m^3^/J
Moisture diffusion	on the basis of Hancox’s equation [22]
Thermal moisture diffusion	2 × 10^−11^ m^2^/(sK)
Moisture transfer coefficient	0.01·× 10^−8^ m/s–all surfaces (150 mm insulation [3])
Mechanical properties	on the basis of experimental tests and considerations reported in [9], with the consideration of concrete hardening temperature by means of an equivalent concrete age
Activation energy	on the basis of experimental tests reported in [25]: 44,178 J/mol - CEM I 42.5R, 40,576 J/mol - CEM II/B-S 32.5R, 43,935 J/mol - CEM II/B-V 32.5R, 35,154 J/mol - CEM III/A 32.5N-LH/HSR/NA, 34,459 J/mol - CEM V/A (S-V) 32.5R-LH/HSR/NA, 31,117 J/mol - VLH V/B (S-V) 22.5

**Table 6:** Analyzed cases.

Slab Thickness	Analyzed Case	Analyzed Results for All Cases
4 m	gravel aggregate and 6 types of cement used in concrete mix for each slab	Temperature development Moisture loss development Development of tensile stresses at top surface of slabs
	basalt/granite/limestone aggregate and CEM III used in concrete mix for each slab	
